# Incidence and risk factors of delirium in multi-center Thai surgical intensive care units: a prospective cohort study

**DOI:** 10.1186/s40560-015-0118-z

**Published:** 2015-12-02

**Authors:** Tanyong Pipanmekaporn, Kaweesak Chittawatanarat, Onuma Chaiwat, Thammasak Thawitsri, Petch Wacharasint, Suneerat Kongsayreepong, THAI-SICU Study Group

**Affiliations:** Department of Anesthesiology, Faculty of Medicine, Chiang Mai University, 110, Intavarorote Rd, Muang District, Chiang Mai, 50200 Thailand; Department of Surgery, Faculty of Medicine, Chiang Mai University, Chiang Mai, 50200 Thailand; Department of Anesthesiology, Faculty of Medicine, Siriraj Hospital, Mahidol University, Bangkok, 10700 Thailand; Department of Anesthesiology, Faculty of Medicine, King Chulalongkorn Memorial Hospital, Bangkok, 10330 Thailand; Department of Anesthesiology, Phramongkutklao Hospital, Bangkok, 10400 Thailand

**Keywords:** Incidence, Risk factor, Surgical intensive care unit, Delirium

## Abstract

**Background:**

Delirium in intensive care units increases morbidity and mortality risk. The incidence and risk factors of delirium vary among studies. This study therefore aimed to determine the incidence and risk factors of delirium in Thai university-based surgical intensive care units.

**Methods:**

A multi-center, prospective cohort study was conducted. All patients who had been admitted to surgical intensive care units (SICU) between April 2011 and January 2012 were enrolled. Delirium was diagnosed using the Intensive Care Delirium Screening Checklists (ICDSC). The univariable and multivariable risk regression analyses were analyzed and presented as risk ratio (RR) and 95 % confidence interval (CI).

**Results:**

The overall incidence of delirium was 3.6 % (162 of 4450, 95 % CI 3.09–4.19 %) whilst the incidences of delirium for patients being admitted ≤48 and >48 h were 0.7 % (21 of 2967, 95 % CI 0.41–1.01 %) and 8.3 % (141 of 1685, 95 % CI 7.04–9.68 %), respectively. The incidence of delirium on each study site was significantly different (range between 0 and 13.9 %, *P* < 0.001). Delirious patients had a significantly higher age (65.3 ± 15.6 versus 61.8 ± 17.3 years, *P* = 0.013), higher Acute Physiology and Chronic Health Evaluation II score (APACHE II score) (16 (12–23) versus 10 (7–15), *P* < 0.001), and higher sequential organ failure assessment score (5 (2–8) versus 2 (1–5), *P* < 0.001). The median duration of delirium was 3 (1–5) days. Delirious patients had significantly longer duration of ICU stay (8 (5–19) versus 2 (1–4), *P* < 0.001) and higher ICU mortality rate (23.5 versus 8.1 %, *P* < 0.001). Sepsis (RR = 3.70, 95 % CI 2.33–5.90, *P* < 0.001), exposure to sedative medications (RR = 3.54, 95 % CI 2.13–5.87, *P* < 0.001), higher APACHE II score (RR = 2.79, 95 % CI 1.98–3.95, *P* < 0.001), thoracic surgery (RR = 1.74, 95 % CI 1.09–2.78, *P =* 0.021), and emergency surgery (RR = 1.70, 95 % CI 1.09–2.65, *P* = 0.019) were independent risk factors of delirium in SICU.

**Conclusions:**

Sepsis, exposure to sedative medications, higher APACHE II score, thoracic surgery, and emergency surgery were independent risk factors of delirium in Thai university-based surgical intensive care units.

## Background

Delirium is defined as a disturbance of consciousness, cognition, attention, and perception, with these symptoms usually acute at the onset and with transient fluctuation over the course of the day [1]. Etiologies of delirium include ischemic brain injury [2], an imbalance of neurotransmitters [3], and peripheral inflammation of the brain [4, 5]. Delirium can lead to several consequences including prolonged mechanical ventilation [4], longer intensive care unit (ICU) admission [4, 5], longer hospitalization [4, 6], higher medical costs [7, 8], higher mortality rate [4, 6], as well as long-term cognitive impairment [4, 6].

The incidences of delirium in the medical and surgical intensive care units (SICU) vary from 11 to 70 % depending on the types of surgical procedures, patient settings, and screening tools for diagnosis of delirium [9–13]. Surgical and medical patients have different pathophysiologies of disease processes and severity of illness; therefore, risk factors of delirium should be determined separately [4]. In addition, different types of surgical procedures could produce various degrees of central nervous system dysfunction and influence the occurrence of postoperative delirium [4]. Surgery is associated with delirium by inducing hypoperfusion [14], microembolism [15], or inflammatory response [16, 17]. Anesthesia also increases risk of delirium. Some medications such as opioids, benzodiazepines, and anticholinergic drugs, which are commonly used for anesthetized patients, are associated with delirium [18]. In addition, anesthesia-related perioperative adverse events including intraoperative hypotension [13], transfusion of blood or blood products [19, 20], and moderate to severe postoperative pain [21] are also associated with postoperative delirium. These predisposing factors therefore may have an influence on the incidence of delirium in SICU, which is different from other ICU settings.

The studied risk factors of delirium in ICU are different among studies. These risk factors include elderly [10], higher Acute Physiology and Chronic Health Evaluation II score (APACHE II score) on admission [9, 22], emergency surgery [22], use of sedative and analgesic medications [12, 22], and trauma [22]. In addition, few studies have focused on risk factors of delirium in SICU [12, 13, 22]. The THAI-SICU study, a large, national multi-center study, recently reported ICU characteristics, overall outcomes, and incidences of adverse events including delirium [23]. The purpose of this study was to specifically determine the incidence and risk factors of delirium in THAI-SICU.

## Methods

The prospective cohort study included patients of nine university-based surgical intensive care units in Thailand. These university SICU were across all regions of Thailand (Siriraj, Ramathibodi, King Chulalongkorn, Phramongkutklao, Sirinthorn Medical Center, Vajira, Maharaj Nakorn Chiang Mai, Srinakharinwirot, and Prince of Songkhla). The recruitment process began after approval was received from the Thai Joint Research Ethics Committee (JREC, no. 001/2011), each institution’s Ethics Committee, and Institutional Review Board. All surgical patients with an age greater than 18 years who were admitted in a SICU between April 2011 and October 2012 were recruited in the study. Patients who had a sustained coma during admission, and required cardiopulmonary resuscitation with no return of spontaneous circulation, and patients who stayed in the ICU for less than 24 h were excluded from the study. In addition, neurosurgical patients and cardiac patients were also excluded because they were not admitted in most sites of SICU depending on individual hospital policies. Patients or their surrogates who met the inclusion criteria were informed about the study in order to obtain their consent. Data collections were divided into three phases including “on admission,” “daily recording data,” and “at discharge.” The criteria for the diagnosis of delirium based on the Intensive Care Delirium Screening Checklists (ICDSC) [24] were discussed by all primary investigators. The assigned intensive care nurses of all sites were trained using ICDSC and completed the scale based on the information from the previous 24 h. All the patients who were included in the study were followed up until being discharged from the ICU. If patients were admitted in the ICU for longer than 28 days, they were followed up until day 28 of the ICU admission. Sedative and analgesic medications were prescribed by the ICU physicians and titrated to achieve the target sedation and analgesic level by bedside nurses. The main outcome of this study was the determination of the incidence and risk factors of delirium. The details of individual patients with delirium included the date of diagnosis, date of recovery, frequency of delirium, diagnostic symptoms of delirium, types of delirium, and medical treatment. All case record forms and documents were further assessed and verified by the central data monitoring unit.

The demographic information included age, gender, comorbidity, American Society of Anesthesiologists physical status (ASA PS), smoking status, emergency surgery, sites of surgery, and the amount of blood and blood product administration. The APACHE II score and the sequential organ failure assessment score (SOFA score) were determined in order to assess the severity of the disease and the patient status, respectively. Other information included presence of sepsis, requirement of mechanical ventilation, laboratory investigation, exposure to sedative and analgesic medications, types of sedative and analgesic medications and route of drug administration, duration of ICU stay (≤48 versus >48 h), hospital stay, and mortality rate.

The statistical analyses were analyzed using STATA, version 11.0 (Stata Corp LP, College Station, TX, USA). The descriptive data are presented as the number and percent for categorical data and mean ± standard deviation, median, and 25 and 75 % percentile for continuous data according to their distribution. Unpaired *t* test, Mann-Whitney *U* test, chi-square test, and Fisher exact probability test were used to detect the difference between the groups in the univariable analysis, as appropriate. The risk factors were analyzed using risk regression with a robust variance estimator and presented as risk ratio and 95 % confidence interval. Any variables which had *P* < 0.20 after the univariable risk regression and all other potential variables associated with the occurrence of delirium were included for the multivariable risk regression. The cut-off point of independent variables including age and serum albumin were determined by the maximum-likelihood estimation method in order to achieve the best discrimination between patients with and without delirium [25]. Sample sizes were calculated based on risk factors of previous studies, and the calculated sample size was 3200 with an accepted *α* of 5 and 80 % power. *P* < 0.05 was considered statistically significant.

## Results

**Fig. 1:**
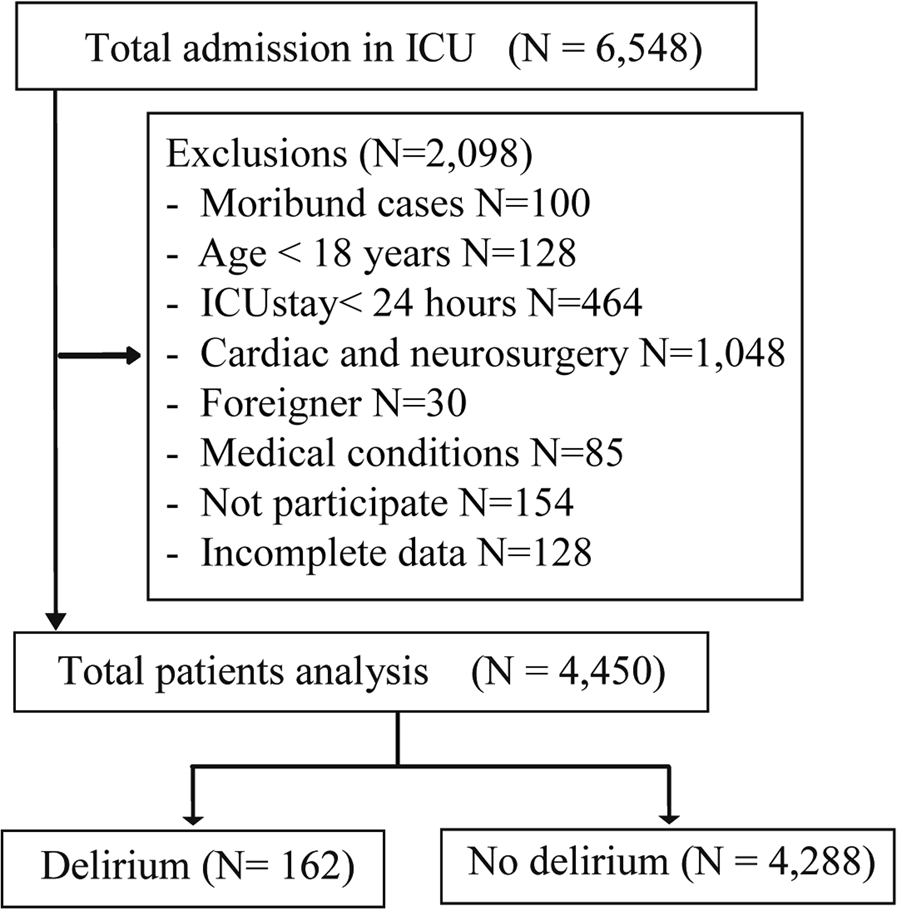
The flow diagram of patients in the study

A total of 6548 patients were admitted to SICU during 19.7 months of recruitment period, and 2098 were excluded. A final number of 4450 patients were included in the analysis. The study flow is presented in Fig. 1. Almost 79 % of patients (3496 of 4450) had a surgical procedure before their ICU admission. The proportion of patients undergoing elective surgical procedures accounted for 69.5 % (2430 of 3496). The median length of ICU admission for patients with elective surgery and emergency surgery were 1 (1–2) days and 3 (2–6) days. The overall incidence of delirium in SICU was 3.6 % (162 of 4450 patients, 95 % CI 3.09–4.19 %). The incidences of delirium were even higher in patients with >48 h admissions than those with ≤48 h (8.3 % (141 of 1685, 95 % CI 7.04–9.68 %) versus 0.7 % (21 of 2967, 95 % CI 0.41–1.01 %), respectively).

Baseline characteristics and outcomes of patients with or without delirium are presented in Table 1. Delirious patients had a significantly higher age (65.3 ± 15.6 versus 61.8 ± 17.3 years, *P* = 0.013), higher APACHE II score (16 (12–23) versus 10 (7–15), *P* < 0.001), and higher SOFA score (5 (2–8) versus 2 (1–5), *P* < 0.001). The incidence of delirium differed by study sites ranging from 0 to 13.9 %. Delirious patients had significantly longer median ventilator days, longer duration of ICU stay, longer hospitalization, and higher ICU and 28-day mortality rate. Details of delirious patients are described in Table 2. Delirium was common on the day of ICU admission (27 of 162, 16.8 %) and the third day (20 of 162, 12.3 %). The most common diagnostic symptoms of delirium included sleep disturbance (6 %), alteration of consciousness (3.1 %), disorientation (2.9 %), and inattention (2.9 %). Types of delirium included mixed (53 %), hyperactive (37 %), and hypoactive (10 %). The percentage of patients who were exposed to midazolam for controlling agitation during ICU admission was about 17 % (740 of 4450). The proportion of patients who received a continuous infusion and intermittent bolus of midazolam was almost similar (50 %).

**Table 1:** Patient characteristics, details of surgery, laboratory investigation, and prognostic outcomes

	Delirium (*n* = 162)	Non delirium (*n* = 4288)	*P*
Variables			
Age (year) mean ± SD	65.3 ± 15.6	61.8 ± 17.3	0.013
<40 (n, %)	11 (6.8)	537 (12.5)	
40–70 (n, %)	76 (47.0)	2175 (50.7)	
>70 (n, %)	75 (46.2)	1576 (36.8)	
Male (*n*, %)	102 (63.0)	2505 (58.4)	0.249
Body mass index (kg/m^2^) (mean ± SD)	22.0 ± 4.6	23.0 ± 5.7	0.031
ASA PS (n, %)			<0.001
I–II	26 (23.6)	1291(39.0)	
III	58 (52.7)	1620 (49.0)	
IV–VI	26 (23.6)	392 (12.0)	
Comorbidity (n, %)			
Hypertension	75 (46.3)	2130 (49.7)	0.399
Diabetes mellitus	33 (20.4)	946 (22.1)	0.610
Coronary artery disease	15 (9.4)	433 (10.1)	0.728
Congestive heart failure	11 (6.8)	93 (2.2)	<0.001
COPD	21 (12.9)	184 (4.3)	<0.001
Chronic renal failure	25 (15.4)	406 (9.5)	0.012
Smoking history (*n*, %)			<0.001
Current smokers	32 (19.7)	498 (11.6)	
Ex-smokers	50 (30.8)	1054 (24.6)	
Non smokers	80 (49.4)	2736 (63.8)	
Numbers of pack years median (P25–75)	15.5( 10–25)	15 (6–30)	0.631
Numbers of operative patients (*n*, %)	119 (73.5)	3377 (79.8)	0.118
Sites of SICU (*n*, %)			<0.001
Site A (incidence 1.9 %)	18 (11.1)	922 (21.5)	
Site B (incidence 1.2 %)	5 (3.1)	404 (9.4)	
Site C (incidence 2.7 %)	15 (9.3)	545 (12.7)	
Site D (incidence 1.9 %)	8 (5.0)	402 (9.3)	
Site E (incidence 0.8 %)	3 (1.8)	390 (9.1)	
Site F (incidence 0 %)	0	139 (3.2)	
Site G (incidence 13.9 %)	109 (67.3)	675 (15.7)	
Site H (incidence 0.8 %)	3 (1.8)	368 (8.6)	
Site I (incidence 0.2 %)	1 (0.6)	443 (10.5)	
Emergency surgery (*n*, %)	79 (66.4)	987 (29.2)	<0.001
Site of surgery (*n*, %)			
Upper abdomen	56 (34.6)	1182 (27.6)	0.051
Lower abdomen	43 (26.5)	1178 (27.5)	0.795
Thoracic	15 (9.8)	162 (3.8)	<0.001
Vascular	5 (3.1)	134 (3.1)	0.978
Extremities	14 (8.6)	371 (8.3)	0.883
Anus	1 (0.62)	52 (1.2)	0.493
Other surgeries	28 (17.2)	1209 (28.2)	0.001
Duration of surgery (min) median (P25–75)	180 (105–275)	240 (150–360)	<0.001
Amount of PRC transfusion (ml) median (P25–75)	100 (0–635)	0 (0–511)	0.274
Amount of FFP transfusion (ml) median (P25–75)	0 (0–780)	0 (0–0)	<0.001
APACHE II median (P25–75)	16 (12–23)	10 (7–15)	<0.001
SOFA median (P25–75)	5 (2–8)	2 (1–5)	<0.001
Sepsis (*n*, %)	109 (67.3)	779 (18.2)	<0.001
Mechanical ventilation (*n*, %)	141 (87.0)	2635 (61.8)	<0.001
Albumin (g/dl) (mean ± SD)	2.4 ± 0.85	2.8 ± 0.80	<0.001
Hemoglobin (g/dl) (mean ± SD)	9.7 ± 2.6	10.6 ± 2.1	<0.001
Blood sugar (mg%) (mean ± SD)	144.0 ± 72.0	164.1 ± 68.8	0.013
PaO2 (mmHg) (mean ± SD)	144.0 ± 72.1	164.1 ± 0.81	0.013
Exposure to sedative drugs (*n*, %)	110 (67.9)	932 (21.7)	<0.001
Exposure to analgesic drugs (*n*, %)	134(82.7)	3683 (85.9)	0.256
ICU admission diagnosis (*n*, %)			0.005
Abdominal	71 (43.8)	1728 (40.3)	
Cardiovascular	31 (19.1)	662 (15.4)	
Respiratory	17 (10.5)	335 (7.8)	
Renal	10 (6.2)	356 (8.3)	
Other	33 (20.4)	1207 (28.2)	
Prognostic outcomes			
Ventilator days (days) median (P25–75)	7 (4–17)	2 (1–4)	<0.001
Length of ICU stay (days) median (P25–75)	8 (5–19)	2 (1–4)	<0.001
Duration of hospital stay (days) median(P25–75)	22 (14–34)	15 (9–26)	<0.001
ICU mortality (*n*, %)	38 (23.5)	349 (8.1)	<0.001
28-day mortality (*n*, %)	45 (27.8)	534 (12.4)	<0.001

Data are presented as mean ± standard deviation (SD), median and 25 and 75 % interquartile range, and number (%)

*ASA PS* American Society of Anesthesiologists physical status, *COPD* chronic obstructive pulmonary disease, *PRC* packed red cell, *FFP* fresh frozen plasma, *APACHE II* Acute Physiology and Chronic Health Evaluation II, *SOFA* sequential organ failure assessment, *ICU* intensive care unit

**Table 2:** Characteristics of delirious patients (*n* = 162)

Factors	Number (percent)
Duration of delirium (days) median (P25–75)	3 (1–5)
Frequency of delirium (*n*, %)	
1	114 (96.6)
2	4 (3.4)
Treatment of delirium	
Benzodiazepine	48 (40.7)
Antipsychotic drugs	40 (34)
α2 agonist	1 (0.8)
Psychiatrist consultation	18 (15.2)
Impaired vision	16 (13.7)
Impaired hearing	13 (11)
History of chronic alcoholism	20 (17.7)
Smoking status	47 (41.6)
Physical restraint	69 (59)
Lab abnormality	
Acidosis	22 (18.8)
Hypoalbuminemia	62 (53)
Received blood or blood product transfusion	62 (55.8)

The data are presented as median and 25 and 75 % interquartile range and number (%)

**Fig. 2:**
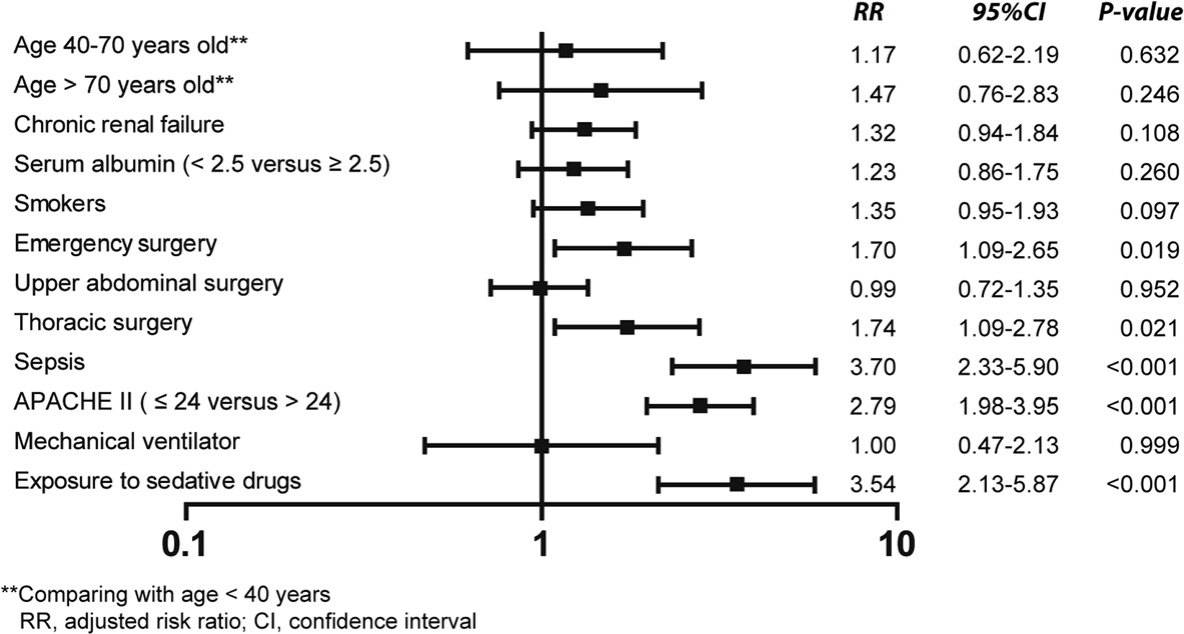
Multivariable risk regression of delirium in surgical intensive care unit. *APACHE II* Acute Physiology and Chronic Health Evaluation II

Age >70 years, smoking, sites of surgical ICU, chronic renal failure, hypoalbuminemia, emergency surgery, thoracic surgery, sepsis, higher APACHE II score, on mechanical ventilator, and exposure to sedative medications increased the risk of postoperative delirium in univariable risk regression (Table 3). In multivariable risk regression analysis, sepsis (risk ratio (RR) = 3.70, 95 % CI 2.33–5.90, *P* < 0.001), exposure to sedative drugs (RR = 3.54, 95 % CI 2.13–5.87, *P* < 0.001), higher APACHE II score (RR = 2.79, 95 % CI 1.98–3.95, *P* < 0.001), thoracic surgery (RR = 1.74, 95 % CI 1.09–2.78, *P =* 0.021), and emergency surgery (RR = 1.70, 95 % CI 1.09–2.65, *P* = 0.019) were independent risk factors of delirium in SICU (Fig. 2).

**Table 3:** Univariable risk regression of ICU delirium

Risk factors	Risk ratio	95 % CI	*P*
Age group (years)			
<40 (n, %)	1.00		Reference
40–70 (n, %)	1.68	0.89–3.16	0.107
>70 (n, %)	2.26	1.20–4.26	0.011
Male	1.20	0.87–1.65	0.259
Smoking	1.77	1.30–2.40	<0.001
Chronic renal failure	1.70	1.11–2.61	0.015
Hypoalbuminemia (<2.5 versus ≥2.5 g/dl)	1.92	1.37–2.67	<0.001
Emergency surgery	4.50	3.08–6.58	<0.001
Upper abdominal surgery	1.37	0.99–1.89	0.056
Lower abdominal surgery	0.95	0.67–1.35	0.799
Thoracic surgery	2.46	1.45–4.91	0.001
Higher APACHE II score (>24 versus ≤24)	1.09	1.07–1.11	<0.001
Sepsis	8.24	5.94–11.45	<0.001
Mechanical ventilation	4.00	2.53–6.29	<0.001
Exposure to sedative medications	7.01	5.05–9.74	<0.001
Exposure to analgesic medications	0.83	0.56–1.25	0.386

*APACHE II* Acute Physiology and Chronic Health Evaluation II

## Discussion

The overall incidence of delirium in SICU patients was 3.6 % in the present study, and this increased up to 8.3 % in patients admitted to SICU for longer than 48 h. However, these incidences were considerably less than those reported by previous studies [9–13]. The majority of patients in this study had a short length of ICU stay (≤48 h), accounted for 67 % of the total population. These patients mainly required postoperative continuous monitoring and critical management for a short period of time. Furthermore, they had significantly lower ASA PS, less comorbidity, and a lower APACHE II score, thereby being at approximately 12 times lower risk for delirium, compared to those with longer than 48 h of ICU stay (incidence 0.7 versus 8.3 %). This corresponds to a previous study showing that extended length of ICU stay almost 8.6 times was associated with increased risk of delirium [4].

The incidences of delirium among sites of surgical ICUs varied between 0 and 14 % (Table 1), and there might be some heterogeneity among these sites during the study period. After exploration of patient characteristics among the sites of SICU, the author found that the site of SICU (site G) which had the highest incidence of delirium had significantly higher percentage of septic patients (31.2 versus 3.3 %) and higher percentage of patients receiving sedative medications (22.1 versus 0.7 %) compared to the site (site F) with the lowest incidence of delirium. Both of these following factors were associated with an increased risk of delirium.

Patients with sepsis significantly had an increased risk of ICU delirium, corresponding to the findings of previous studies [13, 26]. Sepsis can result in acute brain dysfunction and delirium by stimulation of inflammatory cytokine, and endothelial damage, thereby increasing permeability of the blood-brain barrier and causing impairment of capillary blood flow [18, 27]. In addition, these cytokines can cross the blood-brain barrier, increase permeability of the brain tissue, and produce an abnormality of the electroencephalography [18, 28]. Previous studies found that there was an association of delirium and an increased level of serum biomarkers such as neuron-specific enolase or s-100β during severe infection [18, 27]. The knowledge of serum biomarkers helps clinicians understand the relationship between mechanisms and the development of delirium. However, the measurement of these biomarkers is not routinely used in daily practice.

Sedative and analgesic medications are commonly used for critically ill patients for reducing agitation, anxiety, and pain related to surgery and procedure, improving patient and mechanical ventilation synchronization and reducing physiological stress responses [29]. Similar to the results of previous studies, our findings revealed that exposure to sedative medications increased the risk of delirium in ICU almost three times [9, 20, 22, 30]. Also, the incidence of delirium was significantly higher in patients receiving midazolam than those who were not exposed to midazolam (10.3 versus 2.3 %, *P* < 0.001). Benzodiazepine, a gamma aminobutyric acid, can contribute to the occurrence of delirium via several mechanisms including the disturbance of normal sleep pathway, direct impairment of memory function, and alteration of the level of neurotransmitters [31]. According to the current guidelines of the American College of Critical Care Medicine for management of pain and agitation [32], all patients should be frequently assessed for the level of sedation with valid and reliable screening tools in order to maintain an adequate level of depth of sedation during anesthesia and in ICU stay. Providing daily interruption of sedative medication and administration of an alternative sedative drug such as α2 adrenergic agonist are recommended [32].

The APACHE II score represents disease severity of individual patients. Corresponding to the findings of previous studies [9, 30], the present study found that a higher APACHE II score was a strong risk factor of delirium in SICU (RR = 2.12). The authors categorized patients according to their APACHE II score and classified them into two groups by using a cut-off value of 24 before inclusion into the final model during regression analysis. Patients with an APACHE II score higher than 24 indicated high-risk patients. Van Rompaey et al. [20] reported that risk of delirium increased about 2.5 times by univariable regression analysis; however, they could not find any association between the APACHE II score and delirium by multivariable regression analysis. High-risk patients should receive an appropriate medical optimization as well as regular monitoring for delirium.

The present study also found that thoracic surgery increased risk of delirium in ICU. The effect of one lung ventilation during thoracic surgery could increase risk of postoperative delirium by several mechanisms including an activation of pulmonary inflammatory response and cytokines [33], causing several physiological disturbances such as hypoxia or surgical stress [34], and aggravating proinflammatory response [34].

Emergency surgery was an independent risk factor of delirium in ICU in this study. Previous studies also proposed that emergency surgery increased risk of postoperative morbidity and morbidity and increased an incidence of delirium compared to those receiving elective surgery [22, 35]. Although some risk factors such as age [22, 30], mechanical ventilation [20], and opioid exposure [12] have been demonstrated as a risk factor of delirium in ICU, the present study could not find any association between these independent risk factors and delirium.

The present study was the largest, multi-center, prospective cohort study and determined the incidence and risk factors of delirium on surgical ICU in Thailand. The results of this study could be used as a reference and benchmark for Thai academic hospitals, SICU settings in this study. First, this study performed a single delirium assessment per day, which was considerably less than the recommended guideline [32]. This could lead to an underestimation of its incidence due to the fluctuation of symptoms over the course of the day. Secondly, this study did not assess the amount of sedative medications; therefore, we could not demonstrate the causal relationship between sedative medication and delirium. Finally, some potential risk factors of delirium in ICUs such as preoperative cognitive impairment, visual or auditory impairment, and limitations of functional capacity were not included in this study.

## Conclusions

The present study found that sepsis, exposure to sedative medications, higher APACHE II scores, thoracic surgery, and emergency surgery were significant risk factors of delirium in Thai university-based SICU. An identification of high-risk patients, appropriate medical optimization, and careful adjusting of sedative medications according to the level of sedation could reduce an incidence and severity of delirium in ICU.

## Abbreviations

APACHE II score: Acute Physiology and Chronic Health Evaluation II score
ASA PS: American Society of Anesthesiologists physical status
CI: confidence interval
ICDSC: Intensive Care Delirium Screening Checklists
ICU: intensive care unit
JREC: Joint Research Ethics Committee
RR: risk ratio
SICU: surgical intensive care unit
SOFA: sequential organ failure assessment score
